# Expression of Nitric Oxide Synthase and Nitric Oxide Levels in Peripheral Blood Cells and Oxidized Low-Density Lipoprotein Levels in Saliva as Early Markers of Severe Dengue

**DOI:** 10.1155/2021/6650596

**Published:** 2021-02-09

**Authors:** Harsha Hapugaswatta, Ruwani L. Wimalasekara, Suharshi S. Perera, Ranjan Premaratna, Kapila N. Seneviratne, Nimanthi Jayathilaka

**Affiliations:** ^1^Department of Chemistry, Faculty of Science, University of Kelaniya, Kelaniya, Sri Lanka; ^2^North Colombo Teaching Hospital, Ragama, Sri Lanka; ^3^Department of Medicine, Faculty of Medicine, University of Kelaniya, Kelaniya, Sri Lanka

## Abstract

**Background:**

Severe dengue (SD), experienced by only a fraction of dengue patients, can be lethal. Due to the lack of early markers that can predict the evolution of SD, all dengue patients have to be monitored under hospital care. We discovered early oxidative stress markers of SD to identify patients who can benefit from early intervention before the symptoms appear.

**Methods:**

The expression of inducible nitric oxide synthase (iNOS) in peripheral blood cells (PBC), nitric oxide (NO), and oxidized low-density lipoprotein (oxLDL) levels in plasma and saliva collected at early stages of dengue infection from 20 nonsevere dengue fever (DF) patients and 20 patients who later developed SD were analyzed in a retrospective nested case-control study.

**Results:**

The expression of iNOS is significantly (*P* < 0.05) lower in patients who developed SD than in DF patients at admission within 4 days from fever onset. Median plasma NO concentration within 4 days from fever onset is also significantly (*P* < 0.05) lower in patients who developed SD (17.9 ± 1.6 *μ*mol/L) than DF (23.0 ± 2.1 *μ*mol/L). Median oxLDL levels in plasma within 3 days from fever onset is significantly (*P* < 0.05) lower in patients who developed SD (509.4 ± 224.1 ng/mL) than DF (740.0 ± 300.0 ng/mL). Median salivary oxLDL levels are also significantly (*P* < 0.05) lower in patients who developed SD (0.8 ± 0.5 ng/mL) than DF (3.6 ± 2.6 ng/mL) within 4 days from fever onset.

**Conclusions:**

These findings suggest that the expression of iNOS (73% sensitivity, 86% specificity) and plasma NO (96% sensitivity, 61% specificity at 22.3 *μ*mol/L; *P* < 0.05) may serve as early markers of SD within 3 days from fever onset. Salivary oxLDL levels may serve as early noninvasive markers of SD with a sensitivity and specificity, respectively, of 57% and 91% at 0.9 ng/mL; 76% and 55% at 2.3 ng/mL; and 100% and 50% at 4.6 ng/mL (*P* < 0.05) within 4 days from fever onset.

## 1. Introduction

Dengue is an extremely prevalent mosquito-borne viral disease in many tropical countries including Sri Lanka. It is the second most important tropical disease (after malaria) with 284-528 million dengue infections resulting in 67–136 million clinically manifested dengue cases with half the global population at-risk posing a significant public health threat worldwide [1, 2]. Currently, the number of severe dengue (SD) cases in Sri Lanka has dramatically increased. In 2017, 186,101 suspected dengue cases were reported to the Epidemiology Unit of Sri Lanka from all over the island while 2018 only saw 51,659 reported cases of dengue. However, 104,667 cases were reported in 2019 [3]. Most people infected with dengue viruses are asymptomatic while others may suffer a wide range of clinical manifestations from mild fever to SD. Nonsevere dengue fever (DF) is a serious, debilitating condition, and severe manifestations of the disease are major causes of hospitalization and death, globally [1, 4]. Unlike DF, SD is characterized by severe possibly lethal vasculopathy marked with plasma leakage, intrinsic coagulopathy, and massive internal bleeding [5, 6].

Despite the social and clinical impact, there are no antiviral therapies available for the treatment of dengue [7]. The vaccine that is licensed in 18 countries has several limitations because it is only recommended for individuals aged 9-45 years who have had previous exposure to dengue [8]. As such, prevention is mostly limited to vector control measures. Several efficient and relatively reliable diagnostic tests based on PCR or serological testing are available for the detection of dengue virus infections. Rapid lateral flow tests for the presence of dengue NS1 antigen are most commonly administered for dengue diagnosis in Sri Lanka. These diagnostic tests, however, do not distinguish between DF and SD [9]. Limited progress has been made in finding markers that can indicate the evolution of dengue infection to the severe form of the illness at an early stage of infection. In fact, a diagnosis of disease severity is usually made after the patient is presented with SD symptoms. Several studies have compared the transcriptomes of patients that developed DF with those who developed SD to identify molecular markers such as cytokines associated with disease severity [6, 10–16]. In a genome-wide association study, genetic variants in major histocompatibility complex class I polypeptide-related sequence B gene (MICB) and phospholipase C epsilon 1 (PLCE1) have been found to be associated with SD [17]. A later study revealed that these genetic variants are not only associated with SD but also with less severe clinical phenotypes of DF [18]. Allelic forms of MICA and MICB, on the other hand, have been found to strongly associate with susceptibility to illness but not with the severity of infection [19]. Recent studies have also reported differential expression of microRNA in dengue patients and in infected cultured cells [20–24]. However, most of the patient studies do not limit the sample pool to the acute phase of infection at which differential diagnosis is not possible. Thus, despite these efforts, endeavors to discover a prognostic test for SD are yet to become part of the dengue clinical tests, leaving much to be done in finding a solution to this public health crisis.

Inducible nitric oxide synthase (iNOS) has been implicated in the host response to dengue virus infection [25]. The expression of iNOS leads to nitric oxide (NO) biosynthesis resulting in the generation of a highly reactive nitrogen oxide species, peroxynitrite, via a radical coupling reaction of NO with superoxide which in turn causes potent oxidation and nitration reactions of various biomolecules including lipids. NO and oxygen radicals such as superoxide are key molecules in the pathogenesis of various infectious diseases [26]. NO biosynthesis through the expression of iNOS occurs in a variety of microbial infections. iNOS mediated production of nitric oxide, accumulation of reactive oxygen species (ROS), and reactive nitrogen species (RNS), as well as the perturbation of the levels of the intracellular antioxidant; glutathione (GSH) has been reported in DENV-infected human cells and animal models. Elevated lipid and protein oxidation markers in serum and plasma and alteration in the redox status of DENV-infected patients have been associated with increased inflammatory responses, cell death, increased susceptibility to DENV infection, and increased disease severity [27]. iNOS activity and plasma NO have been implicated in inflammatory responses and plasma leakage [25]. SD is characterized by thrombocytopenia (low platelet count) and plasma leakage. Therefore, NO which plays a complex and diverse physiological and pathophysiological role may serve as an early prognostic marker in dengue [28]. A recent study indicates the potential of the levels of serum NO in DF and SD patients as an early marker of disease diagnosis [29]. However, the levels of NO were not evaluated for their potential as early markers of the disease in other biological fluids. Further, the differential expression of iNOS during the early stages of DENV infection among patients who do not present severe symptoms and those who develop severe symptoms has not been evaluated to assess the potential to serve as an early marker of disease severity. *In vitro* studies of DENV and other flavivirus infections such as the Japanese encephalitis virus and West Nile virus suggest that lipids and lipoproteins may play a role in modifying virus infectivity of target cells due to the role of cholesterol-rich lipid rafts in flavivirus entry. In fact, lower total serum cholesterol and low-density lipoprotein cholesterol levels have been shown to associate with subsequent risk of developing severe symptoms of dengue such as dengue hemorrhagic fever/dengue shock syndrome using the WHO 1997 dengue severity classification [30]. Therefore, ROS-mediated oxidation of low-density lipoprotein cholesterol levels may also serve as an early marker of disease severity in dengue. Therefore, in this study, we evaluated whether the severity of dengue infection is correlated with early differential expression of iNOS and resultant changes in NO levels and oxidized low-density lipoprotein cholesterol (oxLDL) levels in plasma from patients who tested positive for dengue within 4 days from fever onset before severe symptoms are presented. Levels of biomarkers such as LDL in saliva have been reported to correlate with serum and plasma LDL levels [31]. Since saliva is a safe and easy to handle biological fluid that can be collected using noninvasive measures, we also evaluated the salivary oxLDL levels in samples from patients presented with symptoms of dengue fever during the early stages of infection compared to those who later developed SD.

## 2. Materials and Methods

### 2.1. Sample Collection and Processing

127 adult male and female patients (above age 18) presented with clinical symptoms of dengue viral infection according to WHO Dengue case classification (2009) (fever, with two of the following criteria: nausea, vomiting, rash, aches and pains, positive tourniquet test, leukopenia with or without warning signs; the warning signs include abdominal pain or tenderness, persistent vomiting, clinical fluid accumulation, mucosal bleeding, lethargy, restlessness, liver enlargement > 2 cm, increase in HCT concurrent with a rapid decrease in platelet count) within 4 days from fever onset who tested positive for onsite NS1 rapid test (SD Bio) were recruited for the study from the North Colombo Teaching Hospital, Ragama, Sri Lanka, from 2015 to 2017 with informed consent. 2.5 mL of blood was collected into EDTA tubes without any fasting schedule, and approximately 500 *μ*L of saliva was collected by spit method from all patients at the time of admission within 4 days from fever onset. The participants were advised to rinse their mouth with water to remove food residue, and the saliva samples were collected 10 minutes after rinsing to avoid sample dilution. The samples were transported and processed at 4°C within an hour from sample collection. Isolated peripheral blood cells (PBC), plasma, and saliva samples (after adding 30 mmol/L NaOH to stabilize the nitrites since nitrites are not stable in acidic medium and saliva can be slightly acidic) were stored at −80°C until sample analysis. A questionnaire was used to collect information pertaining to alcohol consumption, smoking habits, and dietary intake. None of the patients was pregnant. Patients who later developed SD were determined according to the WHO 2009 guidelines based on the evidence of plasma leakage (pleural effusions and ascites detected using a portable bedside ultrasonogram) [32]. Routine blood tests for full blood counts and biochemical tests were carried out every day during the course of hospitalization after admission. None of the patients presented signs of SD at the time of admission within 4 days from fever onset. Twenty patients presented with signs of SD after admission during the course of infection. Samples collected from these patients at the time of admission within 4 days from fever onset were selected as the severe cases for this retrospective nested case-control study. Twenty PBC, plasma, and saliva samples collected at the time of admission within 4 days from fever onset from patients who did not present with signs of SD during the course of infection were randomly selected as controls with DF. No mortality was recorded for the patients recruited for the study.

### 2.2. Ethical Statement

Ethical clearance for patient sample collection was obtained from the ethics review committee of the Faculty of Medicine, University of Kelaniya, Kelaniya, Sri Lanka (reference number-P/119/07/2015). The samples were collected after obtaining informed written consent from all subjects. All subjects voluntarily participated in the study. All methods were carried out in accordance with the protocols approved by the ethics review committee with minimal risk to the study subjects. Patient data and personal information were stored securely. Privacy of personal information was ensured by limiting access to authorized personnel. Clinical data and patient samples were recorded with a serial study number to maintain confidentiality.

### 2.3. Quantitative Real-Time PCR

Gene-specific human primers against the reference gene GAPDH (F primer: 5′ TGCACCACCAACTGCTTAGC 3′, R primer: 5′ GGCATGGACTGTGGTCATGAG 3′, NCBI accession code NM_002046.6, 87 bp) and iNOS (F primer: 5′ CCCCCAGCCTCAAGTCTTATTTC 3′, R primer: 5′ CAGCAGCAAGTTCCA TCT TTCA 3′, NCBI accession code NM_000625.4, 185 bp) were mined from previously published literature [33]. Total RNA was isolated from PBC using miRNeasy serum/plasma kit (Qiagen) with 700 *μ*L QIAzol Lysis buffer and 140 *μ*L chloroform according to the manufacturer's instructions. cDNA was synthesized using the miScript II RT Kit with Hiflex buffer from 12 *μ*L of extracted total RNA according to the product manual (Qiagen). The expression of mRNA was quantified using QuantiTect SYBR Green PCR kit (Qiagen) according to product manual at an annealing temperature of 60°C. Each reaction was carried out in triplicates in 20 *μ*L reaction volume using StepOne real-time PCR Thermal Cycler (Applied Bio). The efficiency of amplification for iNOS was 106%, and GAPDH was 110% based on the standard curve analysis. No-template reactions and melting curve analysis were used to confirm the specificity of target amplification.

The expression levels of iNOS relative to the expression level of the reference gene in DF and SD patients were calculated as 2^−*Δ*Cq^. The relative expression is shown as log_2_ values. Continuous variables were expressed as box and whisker plots with a median for all subjects shown as the centerline, the box representing interquartile range (IQ_25–75_), and the lines showing the range of the data. The fold change of expression was calculated using the equation 2^-*ΔΔ*Cq^ and presented as log_2_ values. A difference in expression based on fold change less than 0.5 between DF and SD cases was considered as downregulation and above 1.5 was considered as upregulation.

### 2.4. Quantification of Plasma and Salivary Nitric Oxide by Griess Reaction

Nitrite content in plasma was measured according to a previously reported method using Griess reaction against a standard series of NaNO_2_ [34]. The plasma sample (60 *μ*L) was deproteinized with 7.5 *μ*L of 200 mmol/L ZnSO_4_ prior to assay for nitrites. The Griess reaction mixture was incubated at room temperature for 20 mins, and absorbance at 540 nm was measured using a Multiskan Go spectrophotometer (Thermo Scientific). Treatment of plasma samples with VCl_3_ to convert nitrates to nitrites followed by colorimetric analysis for nitrites using the Griess reaction did not give higher nitrite reading indicating that there are no detectable nitrates converted to nitrite by this process in the samples. This result was confirmed in 6 plasma samples collected from healthy volunteers. NO is found to almost completely oxidize to nitrite in plasma or other physiological fluids or buffers, where it remains stable for several hours [35]. The same procedure was followed to measure salivary NO.

### 2.5. Quantification of Plasma and Salivary oxLDL by ELISA

oxLDL content in plasma and saliva was measured using human oxLDL ELISA kit (Elabscience) according to the manufactures' protocol with minor modifications. Five microliters of plasma was diluted 1 : 1000 in phosphate-buffered saline (PBS, pH 7), and 25 *μ*L of diluted sample was assayed. 10 *μ*L saliva was assayed in antibody precoated well of micro-ELISA plate after dilution with 15 *μ*L of PBS (pH 7). The oxLDL concentration was calculated based on the concentration series of reference standards of oxLDL provided with the assay kit as follows. The diluted saliva sample was removed from the well, and 100 *μ*L of biotinylated detection antibody was added into the well and incubated for 60 mins at 37°C. The liquid was aspirated, and the well was washed 3 times with wash buffer. Then, 100 *μ*L of horseradish peroxidase conjugate was added into the well and incubated for 30 mins at 37°C. The liquid was aspirated, and 90 *μ*L of substrate reagent was added, washed 5 times with wash buffer, and incubated for 15 mins at 37°C. Then, 50 *μ*L of stop solution was added, and the absorbance was read at 450 nm by using a Multiskan Go spectrophotometer (Thermo Scientific).

### 2.6. Statistical Analysis

q-q plots and Shapiro-Wilk test were used to determine normality at a 95% confidence interval. For the Shapiro-Wilk test, *P* > 0.05 was determined as a normal distribution. Levene's test was used to assess the homogeneity of variance (*P* > 0.05). Statistical significance for differentially expressed targets was determined based on the standard error of the mean (SEM) of ΔC_q_ using an independent *t*-test. Statistically significant differences among the mean ± SD were determined using an independent *t*-test. Statistically significant differences among the median ± median absolute deviation (MAD) was determined using Mann–Whitney *U* test for nonparametric independent samples. *P* < 0.05 was considered statistically significant. Logistic regression analysis for odds ratio, receiver operator characteristics, the area under the curve, specificity, and sensitivity were determined using IBM SPSS Statistics, 2013 version at a 95% confidence interval.

## 3. Results

### 3.1. Clinical Characteristics at Admission

**Figure 1:**
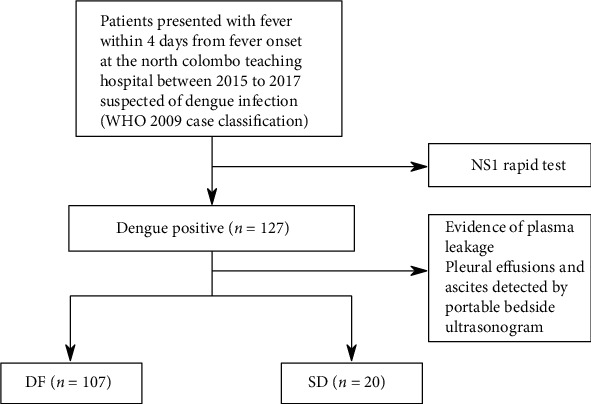
Participant flow diagram.

Samples were collected at admission from patients recruited on day 2 (*n*_DF_ = 2, *n*_SD_ = 3), day 3 (*n*_DF_ = 6, *n*_SD_ = 12), and day 4 (*n*_DF_ = 12, *n*_SD_ = 5) from fever onset. Therefore, there were 8 DF and 15 SD samples collected within 3 days from fever onset. Most of the subjects were male (77%), with a median age of 30 (18-60), while the female subjects had a median age of 24 (19-60) years. The participant flow diagram of the study is given in Figure 1. At admission, there were no statistically significant differences in median laboratory clinical parameters such as thrombocytopenia, leukopenia, hematocrit count, and AST and ALT levels in patients who later developed SD compared with the randomly selected DF patients included in the analysis (Table 1). These findings are consistent with the clinical parameters at admission for all patients who tested positive for dengue NS1 antigen (Supplementary Table 1). Therefore, it was not possible to make a prognosis of SD based on clinical characteristics at the time of admission within 4 days from fever onset.

### 3.2. iNOS Expression in Dengue Patients within Four Days from Fever Onset

**Figure 2:**
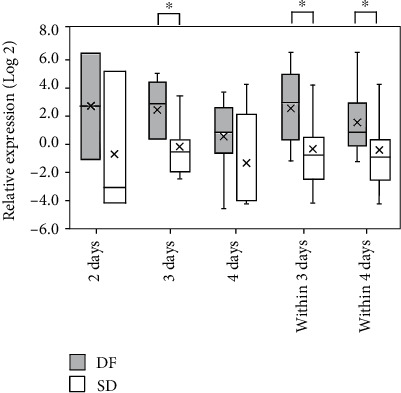
Expression of iNOS in PBC from DF patients and patients who later developed SD. Relative expression of iNOS in patient samples collected at admission from patients recruited on day 2, day 3, day 4, within 3 days, and within 4 days from fever onset designated as DF and SD. Relative expression based on 2^-*Δ*Cq^ values against GAPDH presented as log values to the base 2. ^∗^*P* < 0.05 based on ΔCq ± SEM using independent *t*-test.

Since iNOS has been implicated in the host response to dengue virus infection, the level of iNOS expression was analyzed in PBC collected at the time of admission within 4 days of fever onset. The data were determined to be normally distributed. SD patients showed significantly (*P* < 0.05) lower iNOS expression compared to the DF patients within 4 days from fever onset. Furthermore, iNOS expression in SD patients admitted on day 3 from fever onset was also significantly (*P* < 0.05) low compared to that of DF patients (Figure 2, Supplementary Figure 1).

Logistic regression analysis of iNOS expression within 3 days from fever onset is predictive of SD (odds ratio, 1.74; 95% CI 1.02-2.98; *P* < 0.05) with an area under the receiver operating curve of 0.77. The sensitivity and specificity for the development of SD were 0.73 and 0.86, respectively, at ΔCq for iNOS expression of -0.09.

### 3.3. Level of Nitric Oxide in Plasma and Saliva from Acute Dengue Patients

**Figure 3:**
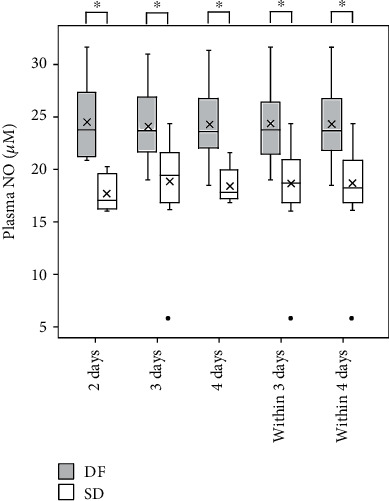
NO in plasma collected at admission within 4 days of fever onset from DF patients and patients who later developed SD. Plasma NO levels in patient samples collected on day 2, day 3, day 4, within 3 days, and within 4 days from fever onset. Data expressed as box and whisker plots with median for all subjects shown as the center line, the box representing interquartile range (IQ_25–75_), and the lines showing the range of the data. ^X^Mean, °outlier > 1.5 times interquartile range beyond the quartiles, ^∗^*P* < 0.05.

The expression of iNOS results in NO biosynthesis. We quantified the levels of NO in plasma using the Griess reaction. Preliminary measurement of NO by the Griess reaction after conversion of nitrate to nitrite revealed that there was no detectable level of nitrate in the plasma samples. Therefore, the nitrite levels as measured by the Griess reaction was taken as the total NO in the plasma samples. Median plasma NO concentration at admission within 4 days from fever onset in patients who later developed SD (17.9 ± 1.6 *μ*mol/L) is significantly (*P* < 0.05) lower than that of DF group (23.0 ± 2.1 *μ*mol/L). A significant decrease in median NO levels (*P* < 0.05) in the SD patients was observed in samples collected at admission on day 2, day 3, day 4, and within 3 days from fever onset (Figure 3). The data are not normally distributed. Therefore, Mann–Whitney *U* test was used to determine significant differences.

Logistic regression analysis of plasma NO level within 4 days from fever onset was found to be predictive of SD (odds ratio, 0.54; 95% CI 0.40-0.72; *P* < 0.05) with an area under the receiver operating curve of 0.89. The sensitivity and specificity for the development of SD within 4 days from fever onset were 0.90 and 0.70, respectively, at plasma NO level of 21.3 *μ*mol/L (*P* < 0.05).The plasma NO level on day 3 and within 3 days from fever onset is also predictive of SD (odds ratio, 0.53; 95% CI 0.30-0.94;*P* < 0.05) with an area under the receiver operating curve of 0.87, sensitivity of 0.95, and specificity of 0.72 at plasma NO level of 22.2 *μ*mol/L (*P* < 0.05and odds ratio, 0.49; 95% CI 0.30-0.81;*P* < 0.05) with an area under the receiver operating curve of 0.90, sensitivity of 0.96, and specificity of 0.61 at plasma NO level of 22.3 *μ*mol/L (*P* < 0.05), respectively. Salivary NO levels among the patients fluctuate in a wide range in both study groups. Therefore, no significant differences were observed between the groups.

### 3.4. Oxidized LDL Levels in Plasma and Saliva

**Figure 4:**
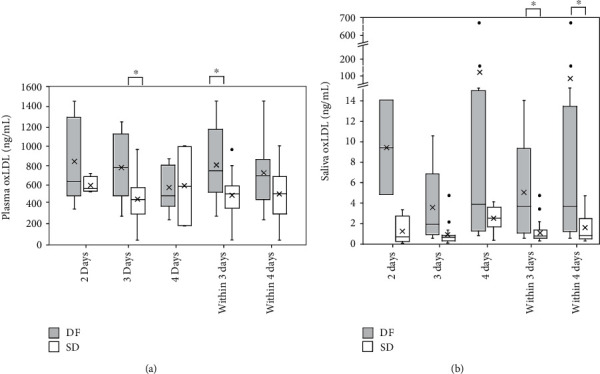
oxLDL in plasma and saliva from DF patients and patients who later developed SD collected at admission within 4 days from fever onset. oxLDL levels in (a) plasma and (b) saliva collected at admission on day 2, day 3, day 4, within 3 days, and within 4 days from fever onset. Data expressed as box and whisker plots with median for all subjects shown as the center line, the box representing interquartile range (IQ_25–75_), and the lines showing the range of the data. ^X^Mean, °outlier > 1.5 times interquartile range beyond the quartiles, ^∗^*P* < 0.05.

L-arginine-NO pathway is involved in the effects of ox-LDL on platelet function [36]. Therefore, plasma oxLDL levels were analyzed at admission within four days from fever onset using ELISA. Median plasma oxLDL concentration at admission within 4 days from fever onset in patients who later developed SD (509.4 ± 179.7 ng/mL) is lower than that of DF patients at admission (688.8 ± 213.4 ng/mL). However, this difference was not statistically significant. The median oxLDL levels in the SD group (509.4 ± 224.1 ng/mL) showed a significantly (*P* < 0.05) low oxLDL levels in plasma collected within 3 days from fever onset compared to patients who did not develop SD (740.0 ± 300.0 ng/mL). Although the sample numbers in each group were low, the oxLDL levels in the SD group were also lower in samples collected at admission on day 2 from fever onset and significantly lower (*P* < 0.05) on day 3 (SD = 451.9 ± 115.3 ng/mL; DF = 783.1 ± 249.4 ng/mL (Figure 4(a)).

The adjusted odds ratio, as calculated by logistic regression analysis of plasma oxLDL level after adjustment for number of days from fever onset, was not predictive of SD within 3 days from fever onset (0.99; 95% CI 0.99-1.00, *P* < 0.05). However, at plasma oxLDL cutoff value of 603.8 ng/mL within 3 days from fever onset, the area under the receiver operating curve is 0.76. The sensitivity and specificity for the development of SD are 0.81 and 0.77, respectively (*P* < 0.05).

oxLDL levels in saliva samples collected at admission within 4 days from fever onset from patients with positive diagnosis for dengue were analyzed using ELISA to evaluate whether the differences observed in the plasma samples were detectable in saliva samples as well. Median salivary oxLDL in SD patients at admission, within 4 days from fever onset (0.8 ± 0.5 ng/mL) is significantly lower (*P* < 0.05) than that of DF patients (3.6 ± 2.6 ng/mL). The median salivary oxLDL at admission, within three days from fever onset in SD patients (0.7 ± 0.3 ng/mL), is also significantly lower (*P* < 0.05) than that of DF patients (3.6 ± 2.6 ng/mL) suggesting that salivary oxLDL may also serve as an early noninvasive marker for SD (Figure 4(b)).

Logistic regression analysis of salivary oxLDL levels at admission within 4 days from fever onset was found to be predictive of SD (odds ratio, 0.69; 95% CI 0.49-0.97, *P* < 0.05) with an area under the receiver operating curve of 0.75. The sensitivity and specificity for the development of SD were 0.57 and 0.91, respectively, at salivary oxLDL level of 0.9 ng/mL; 0.67 and 0.58, respectively, at salivary oxLDL level of 2.0 ng/mL; 0.76 and 0.55, respectively, at salivary oxLDL level of 2.3 ng/mL; and 1.00 and 0.50, respectively, at salivary oxLDL level of 4.6 ng/mL (*P* < 0.05).

## 4. Discussion

iNOS expression in PBC, plasma NO, plasma oxLDL, and salivary ox LDL levels show significant differences between the DF patients and patients who later developed SD during the acute phase of infection. In the present study, patients who developed severe symptoms show significantly (*P* < 0.05) lower levels of iNOS expression in PBC and a corresponding decrease in the plasma NO levels compared to those who did not develop severe symptoms during the early phase of infection. This may be due to the role of iNOS and NO as part of the host defense mechanism which appears to be compromised even during the early phase of infection in the individuals who later developed SD.

The observed decrease in the NO levels in SD patients during the acute phase of infection was significant within 2 days from fever onset and remained significantly low in plasma during the acute phase of infection. These findings are consistent with the reported differences of serum NO and nitrite levels suggesting their role as early marker of disease severity for dengue [29]. However, the plasma NO levels are at least four fold higher than serum NO levels with a clear distinction of the levels between DF patients and patients who later developed SD with an odds ratio of approximately 0.5 for samples collected on day 3, day 4, and within 3 days and 4 days from fever onset, indicating that plasma NO may serve as a more robust marker.

Oxidation of lipids by NO can result in increased levels of plasma oxLDL which promotes vasoconstriction and platelet activation with alterations in platelet function which has been connected to dengue-associated plasma leakage [36, 37]. Low plasma oxLDL levels corresponding to the low plasma NO was observed at admission within 4 days from fever onset in dengue patients who later developed SD. Therefore, the plasma oxLDL levels do not appear to participate in the development of symptoms of SD such as plasma leakage during the early phase of infection.

Similarly, salivary oxLDL levels showed a significant decrease in the patients who later developed SD compared to the DF patients within 4 days of infection proving to be an excellent noninvasive biological source for predictive markers for dengue. Salivary oxLDL levels have been shown to correlate with the serum oxLDL levels [31]. oxLDL has also been associated with cardiovascular risks [31]. Therefore, oxLDL levels in a larger cohort of dengue patients during acute phase of infection may be necessary to validate the role of oxLDL in saliva as a noninvasive early prognostic biomarker of SD and evaluate the effect of the confounding factors. Although saliva may serve as a noninvasive source for NO levels, saliva was proven to be an unreliable biological source due to high standard deviation of NO concentration that may be resulting from the influence of oral health and diet [38].

Our study is limited by the relatively small sample sizes for 2, 3, and 4 days from fever onset and relatively few samples from female patients to assess the potential of these markers to predict the outcome within these parameters. Therefore, further analysis in a larger cohort within each day from fever onset during the acute phase is needed to assess the full potential of these biomarkers to distinguish DF patients from those who progress to SD during the early stages of infection. We were unable to determine whether dietary habits, social habits such as smoking, and noncommunicable conditions such as high cholesterol and diabetes influence the iNOS expression, plasma NO, and plasma and salivary oxLDL among the DF and SD patients within four days from fever onset, due to unreliable response rate from the subjects. Further, area under the receiver operating curve in the case of salivary oxLDL levels is relatively low (only 0.75). Therefore, further analysis in a larger cohort within each day from fever onset during the acute phase is needed to assess the full potential of oxLDL as a noninvasive biomarker to distinguish DF patients from those who progress to SD during the early stages of infection.

Identifying early molecular markers of SD may help distinguish dengue patients who would benefit from early intensive therapy and hospitalization before severe symptoms appear and increase the availability of public health resources and also mitigate the cost of public health and the impact on the national economy.

## 5. Conclusions

Differential expression of iNOS, plasma NO, and salivary oxLDL levels may serve as reliable early biomarkers to predict the development of SD within 4 days from fever onset. Our findings also suggest saliva as a potentially new noninvasive biological source for early prognosis of disease outcome for dengue.

## Figures and Tables

**Table 1:** Clinical characteristics of dengue patients and samples collected for analysis at the admission (median ± MAD).

	DF patients (*n* = 20)	SD patients (*n* = 20)	*P* values
Gender (male %/female %)	68/32	85/15	
Age (years)	34 (18-60)	29 (19-60)	0.75
Platelet (×1000 cells/mm^3^)	113.0 ± 51.8	125.0 ± 37.2	0.57
Hematocrit (%)	39.7 ± 4.6	39.7 ± 4.0	0.98
Hemoglobin (g/dL)	13.7 ± 2.5	13.0 ± 2.8	0.31
White blood cells (×1000 cells/mm^3^)	3.5 ± 40.1	4.6 ± 60.2	0.39
Neutrophils (%)	64.1 ± 18.9	74.5 ± 19.9	0.19
Lymphocytes (%)	27.60 ± 12.8	16.0 ± 13.7	0.08
Eosinophils (%)	0.6 ± 6.1	1.0 ± 2.2	0.88
AST (U/L)	34.0 ± 39.7	56.0 ± 29.0	0.29
ALT (U/L)	37.1 ± 31.1	43.0 ± 24.2	0.43

## Data Availability

The data used to support the findings of this study are included within the article and the supplementary files.

## Supplementary Materials

Supplementary Materials Supplementary Table 1: Clinical characteristics of dengue fever and severe dengue fever patients at the admission. Supplementary Figure 1: Fold change of iNOS expression in PBC between DF and SD patients at admission recruited on, day 2, day 3, day 4, within 3 days, and within 4 days from fever onset.
