# Supplementary material for: Expression of Nitric Oxide Synthase and Nitric Oxide Levels in Peripheral Blood Cells and Oxidized Low-Density Lipoprotein Levels in Saliva as Early Markers of Severe Dengue
Source: Biomed Res Int. 2021 Feb 9;2021:6650596. doi: 10.1155/2021/6650596 (PMC7889359; doi:10.1155/2021/6650596)
Supplement: Supplementary Materials — Supplementary Table 1: Clinical characteristics of dengue fever and severe dengue fever patients at the admission. Supplementary Figure 1: Fold change of iNOS expression in PBC between DF and SD patients at admission recruited on, day 2, day 3, day 4, within 3 days, and within 4 days from fever onset. [file 6650596.f1.zip › Supplementary Figure 1.docx]

*

*

*

Supplementary Figure 1: Fold change of iNOS expression in PBC between DF and SD patients at admission recruited on, day 2, day 3, day 4, within 3 days and within 4 days from fever onset. iNOS expression was normalized against GAPDH and 2^-ΔΔCq^ values presented as log to the base 2. *P < 0.05 (independent t – test).
