# Supplementary material for: Expression of Nitric Oxide Synthase and Nitric Oxide Levels in Peripheral Blood Cells and Oxidized Low-Density Lipoprotein Levels in Saliva as Early Markers of Severe Dengue
Source: Biomed Res Int. 2021 Feb 9;2021:6650596. doi: 10.1155/2021/6650596 (PMC7889359; doi:10.1155/2021/6650596)
Supplement: Supplementary Materials — Supplementary Table 1: Clinical characteristics of dengue fever and severe dengue fever patients at the admission. Supplementary Figure 1: Fold change of iNOS expression in PBC between DF and SD patients at admission recruited on, day 2, day 3, day 4, within 3 days, and within 4 days from fever onset. [file 6650596.f1.zip › Supplementary Table 1.docx]

Supplementary Table 1: Clinical characteristics of dengue fever and severe dengue fever patients at the admission (Median ± MAD).

|  | DF (n=94) | SD (n=33) | P value |
| --- | --- | --- | --- |
| Gender (Male%/Female%) | 71/29 | 76/24 |  |
| Age | 24 (18-60) | 24 (19-60) |  |
| Platelet (X1000 cells/mm^3^) | 135.5±36.5 | 137.0±38.0 | 0.90 |
| Hematocrit (%) | 40.0±3.3 | 39.7±2.3 | 0.91 |
| Hemoglobin (g/dl) | 13.8±1.2 | 13.0±0.8 | 0.10 |
| White blood cells (X1000 cells/mm^3^) | 3.7±1.1 | 4.6±1.1 | 0.76 |
| Neutrophil (%) | 64.3±11.5 | 72.2±10.2 | 0.08 |
| Lymphocytes (%) | 26.9±9.9 | 16.0±8.3 | 0.40 |
| Eosinophil (%) | 0.9±0.8 | 1.0±1.0 | 0.82 |
| AST (U/l) | 38.0±16.0 | 58.5±20.5 | 0.10 |
| ALT (U/l) | 35.8±15.8 | 43.2±13.8 | 0.15 |
